# When herpes masks a greater threat: A case report of granulomatosis with polyangiitis

**DOI:** 10.1097/MD.0000000000042609

**Published:** 2025-05-30

**Authors:** Shu-Qing Ma, Jiantao Wu, Naishi Wu, Hanlong Guo, Qu-Cheng Huang, Wen-Juan Liao

**Affiliations:** a Department of Pulmonary and Critical Care Medicine, Renmin Hospital of Qingxian, Cangzhou, China; b Cardiovascular Surgery Department, Qingdao Hospital, University of Health and Rehabilitation Sciences (Qingdao Municipal Hospital), Qingdao, China; c Department of Cardiovascular Surgery, Tianjin Medical University General Hospital, Tianjin, China; d Department of The First Clinical Medical College, Gannan Medical University, Ganzhou, China; e Department of Child, The First Affiliated Hospital with Gannan Medical University, Xinxiang, China.

**Keywords:** anti-neutrophil cytoplasmic antibody, granulomatosis with polyangitis, hematuria, nasal nodules, respiratory failure, skin herpes

## Abstract

**Rationale:**

Granulomatosis with polyangiitis (GPA) often involves the respiratory tract and kidneys. However, it can also affect other organs, as noted in sporadic case reports, complicating the diagnostic process. To date, few cases of GPA have been reported that were discovered through skin herpes and rapidly developing lesions in multiple organs, including the eyes, nose, respiratory tract, lungs, and kidneys. This condition can lead to significant mortality if not diagnosed and treated promptly.

**Patient concerns:**

A 19-year-old girl was seeking medical advice for herpes lesions on her torso and extremities. She developed gum swelling, which was later determined to be secondary to a misdiagnosis of chickenpox. After treatment with topical medication, she experienced conjunctival congestion and partial loss of vision. A few days later, she rapidly developed dyspnea and fever and was admitted to the hospital for further treatment.

**Diagnoses:**

Following a multidisciplinary team discussion, vasculitis-associated pneumonia was suspected. Finally, diseased tissue was also found in her nasopharynx, which was confirmed by tissue biopsy and laboratory tests as GPA.

**Interventions:**

She was treated with drugs such as methylprednisolone, prednisone, and cyclophosphamide.

**Outcomes:**

Her condition was brought achieved controlled remission, chest computed tomography reexamination showed that the shadows in the patient’s lungs had become smaller and lighter.

**Lessons:**

It has been highlighted that GPA is a complex disease with a wide range of clinical manifestations, requiring dermatologists, respiratory specialists, ophthalmologists, otolaryngologists, rheumatologists, and other medical professionals to have a deep understanding of this condition. Understanding the clinical characteristics, associated risks, and treatment outcomes is crucial for improving patient care. This report adds information on the clinical features of GPA, paving the way for diagnostic and therapeutic strategies.

## 1. Introduction

Anti-neutrophil cytoplasmic antibody (ANCA)-associated vasculitis (AAV) is a systemic autoimmune disease that may be caused by genetic, environmental, and immune factors. It is a group of systemic vasculitides associated with small-caliber vessel necrotizing vasculitis, characterized by the detection of ANCAs in serum. AAV mainly involves small blood vessels (arterioles, tiny arteries, tiny veins, and capillaries) but can also affect medium-sized arteries; this is the most common type of systemic small vessel vasculitis in clinical practice.^[1,2]^ Classic AAVs include granulomatosis with polyangiitis (GPA), microscopic polyangiitis, and eosinophilic granulomatosis with polyangiitis, as well as drug-induced AAV.^[2,3]^ Previous reports have shown that this disease is characterized by damage to the upper respiratory tract, lungs, and kidneys, with very few patients exhibiting lesions in the skin, gingiva, eyes, and nose.^[4–7]^

## 2. Case report

**Figure 1.**
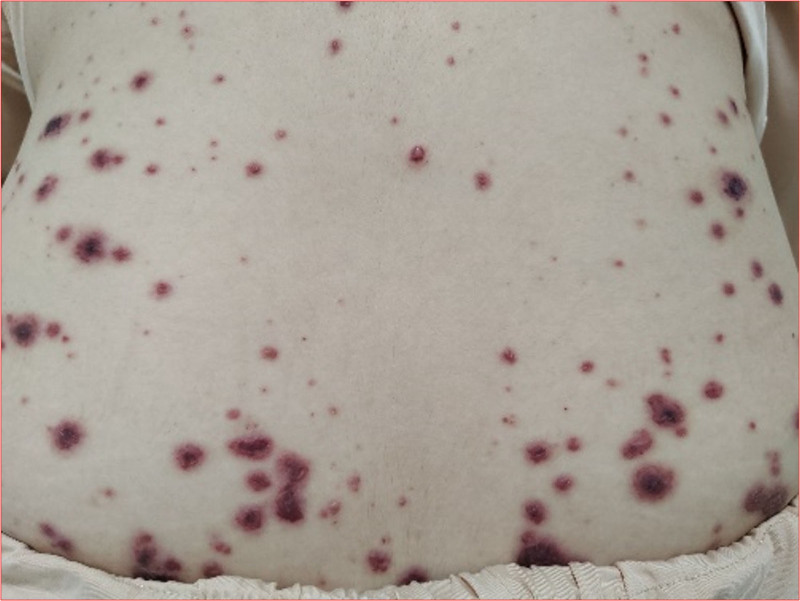
Several skin areas were covered with herpes on the patient’s lower back.

**Figure 2.**
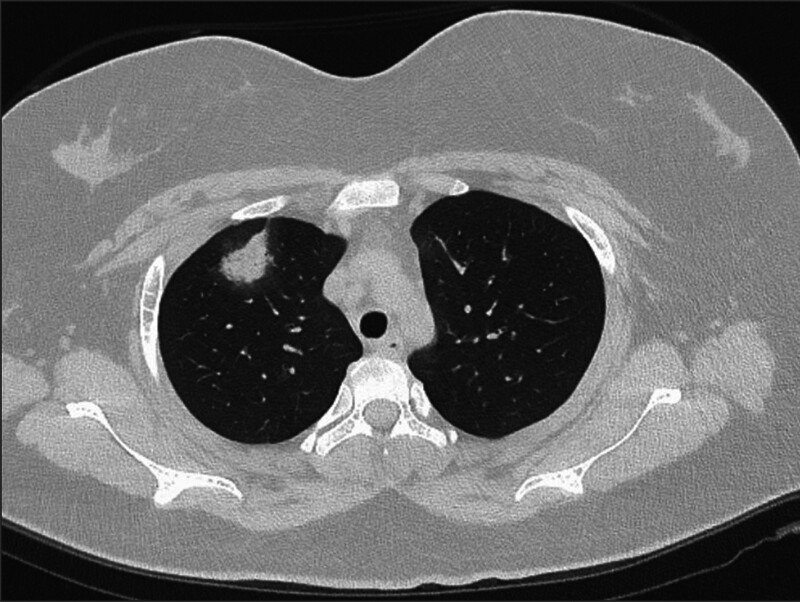
Sixteen-slice computerized tomography images of the chest showed localized patchy shadows in the superior lobar of the right lung.

A 19-year-old girl was seeking medical advice for multiple lesions of herpes without an obvious cause (Fig. 1). The community physician diagnosed chickenpox and administered acyclovir orally, along with calamine lotion. After 3 days, the girl showed no improvement in her symptoms and developed swollen gums. The community doctors provided oral hygiene treatment and prescribed oral metronidazole. Two days later, her symptoms had not eased, and she exhibited conjunctival congestion. Retinal photography indicated a significant decrease in visual acuity, a blurred optic disc boundary, and fluorescence leakage. The ophthalmologist recommended hospitalization and further examination, but she refused. Two days later, she developed a fever, dyspnea, hematuria, a temperature of up to 39.6 °C, and occasional cough with blood-tinged sputum. During the course of the disease, she experienced intermittent epistaxis, which was noticeable when she blew her nose. Throughout the illness, the patient reported no chest pain, palpitations, abdominal pain, diarrhea, acid reflux, vomiting, frequent urination, urgent urination, or dysuria. She had no history of specific infectious diseases and no history of pollen or pet contact. She denied having hereditary diseases in her family. She underwent a nasopharyngoscopy, which revealed herpes hyperplasia nodules in the soft palate and posterior pharyngeal wall. While awaiting the pathological results of these nodules, the patient progressed to respiratory failure and was placed on a noninvasive ventilator to assist with breathing. Subsequently, the patient underwent a chest computed tomography, which revealed multiple localized patchy shadows in the right lung (Fig. 2).

On the multidisciplinary discussion team, tuberculosis, bacterial, viral, and fungal infections, allergy, IgG4-related pulmonary disease, Behcet disease with pulmonary lesions, IgA nephropathy, anaphylactoid purpura, and ANCA-associated pulmonary disease are being considered. Then, laboratory testing for these diseases began immediately and includes the tuberculin skin test, T-cell spots for tuberculosis infection, metagenomic next-generation sequencing of skin herpes exudates (including viruses, bacteria, and fungi), ANCA test, serum IgG4 concentration test, intradermal patterning test or femoral vein thickness measurement, and qualitative urinary protein test, as well as the inclusion and exclusion of the diagnosis of anaphylactoid purpura.

**Figure 3.**
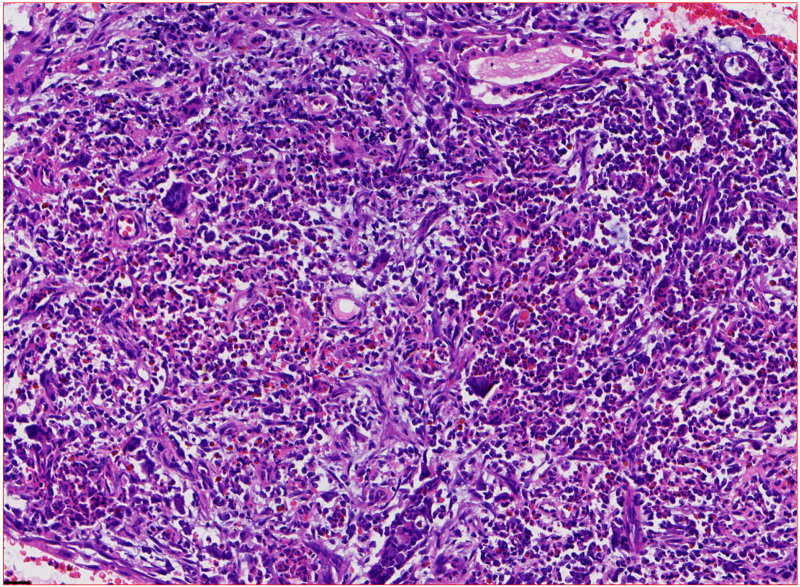
Pathological examination of the nasal herpes revealed multinucleated giant cells with granulomatous changes (HE, 40×).

**Figure 4.**
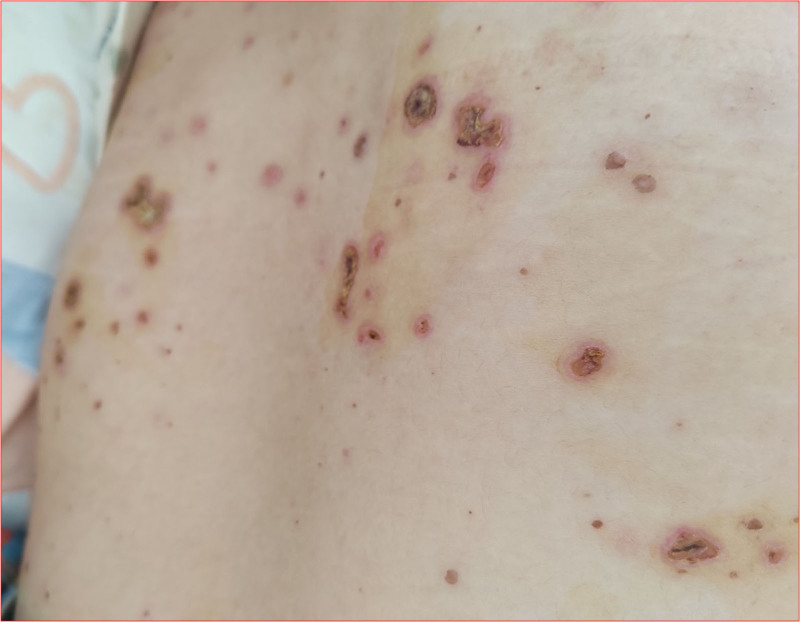
Image of skin herpes was significantly reduced in the lower back.

**Figure 5.**
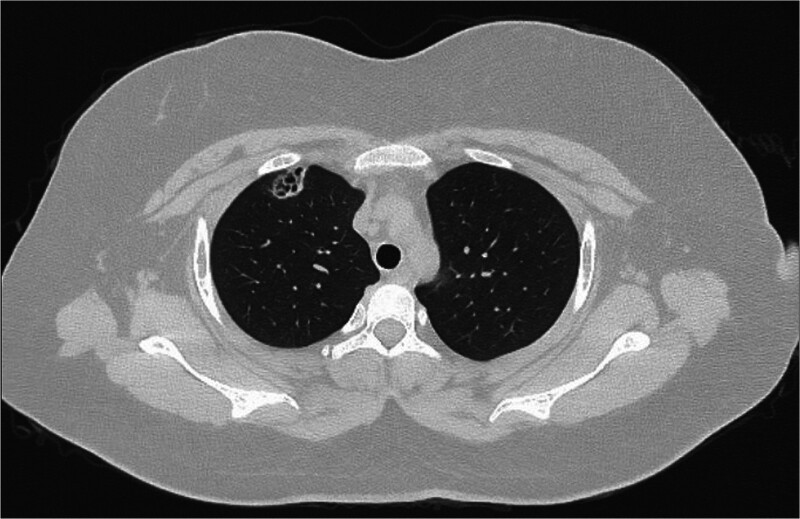
Sixteen-slice computerized tomography reexamination images of the chest showed that the shadow of the patient’s lungs became smaller and lighter in the superior lobar of the right lung.

The most significant results showed that the C-ANCA and proteinase-3 (PR3)-ANCA tests were positive according to the immune profile, and additional laboratory test results are presented in Table 1. Pathology of the nasal nodules revealed granulomatous inflammatory changes (Fig. 3). Finally, she was diagnosed with GPA based on the diagnostic criteria established by the American College of Rheumatology. The patient received steroid pulse therapy (intravenous methylprednisolone, 1000 mg for 5 days), followed by oral prednisone (80 mg/day) and cyclophosphamide (0.75 g/m²). The skin improved rapidly (Fig. 4), and occult blood and proteinuria disappeared. Chest computed tomography reexamination showed that the shadows in the patient’s lungs had become smaller and lighter (Fig. 5). Two weeks after discharge, the patient’s symptoms improved further, and her vision almost returned to normal. So, she expressed great satisfaction with the diagnosis and treatment of the medical care received in the hospital.

**Table 1:** Patient laboratory test results.

Laboratory tests	Normal range	Results
WBC (×10^9^/L)	4–10	12.67
Serum amyloid A (mg/L)	<10	350
C-reactive protein (mg/L)	<10	350
Albumin (g/L)	35–55	33.9
Alanine aminotransferase (U/L)	7–40	34
Aspartate aminotransferase (U/L)	13–35	11
Direct bilirubin (µmol/L)	0–6.8μmol/L	7.7
Creatinine (µmol/L)	41–81	54.7
Urea nitrogen (µmol/L)	3.6–9.5	3.72
Erythrocyte sedimentation rate (mm/h)	0–20	128
Protein (urinalysis routine)	Negative	3+
Urobilinogen (urinalysis routine)	Negative	3+
Occult blood (urinalysis routine)	Negative	3+
Anti-nRNP/Sm antibody	Negative	Negative
Anti-Sm antibody	Negative	Negative
Anti-SSA antibody	Negative	Negative
Anti-Ro52 antibody	Negative	Negative
Anti-Scl-70 antibody	Negative	Negative
Anti-Jo-1 antibody	Negative	Negative
Anti-CENP B antibody	Negative	Negative
Anti-PCNA antibody	Negative	Negative
Anti-ds DNA antibody	Negative	Negative
Anti- nucleosome antibody	Negative	Negative
Anti-histone antibody	Negative	Negative
Anti-rRNP antibody	Negative	Negative
Anti-AMA M2 antibody	Negative	Negative
Anti-proteinase-3 antibody	Negative	Positive
Anti-myeloperoxidase antibody	Negative	Negative
Anti-neutrophil cytoplasmic antibody-pANCA	Negative	Negative
Anti-neutrophil cytoplasmic antibody-cANCA	Negative	Positive
Serology (colloidal gold-labeled technique)	Negative	Negative

## 3. Discussion

Most AAV patients have systemic symptoms such as fever, fatigue, loss of appetite, and weight loss.^[2]^ Early GPA lesions are sometimes limited to a certain part of the upper respiratory tract and are often misdiagnosed. Among the 3 AAVs, GPA was most frequently involved in the ear–nose–throat, upper respiratory tract, and lungs.^[2]^ The kidney was involved mainly in early microscopic polyangiitis.^[2]^ Early eosinophilic granulomatosis with polyangiitis is characterized by allergic bronchial asthma, peripheral blood eosinophilia, fever, and pulmonary infiltration.^[2]^ Cutaneous manifestations are also frequently observed in GPA, with patients presenting with a variety of skin lesions such as palpable purpura, mucocutaneous ulcers, and subcutaneous nodules. These skin findings can sometimes be the initial clue to the underlying vasculitic process and may correlate with systemic involvement, particularly renal disease.^[8]^ The disease may mimic conditions such as pyoderma gangrenosum, presenting with cutaneous ulceration. While ANCA are positive in approximately 90% of GPA cases, their absence does not exclude the diagnosis.^[9]^ Therefore, clinicians should maintain a high suspicion for GPA in patients with skin lesions and respiratory tract involvement, even when ANCA is negative.

Ocular involvement in GPA is uncommon and can present in various forms, such as retinal vasculitis, anterior necrotizing scleritis, or compressive optic neuropathy due to retrobulbar inflammatory mass. These ocular manifestations can be severe and may serve as initial indicators of the disease. In some cases, the diagnosis of GPA is made based on ocular symptoms, highlighting the importance of recognizing these signs early in the disease course.^[10]^ Nasal involvement is one of the most common features of GPA, often presenting as chronic rhinosinusitis. The nasal mucosa can exhibit crusting, friable erythematous mucosa, and granulation. In severe cases, cartilage destruction can lead to a saddle-nose deformity.^[7]^

The pathophysiology of GPA involves the production of ANCA, particularly those targeting PR3. These antibodies play a significant role in the disease’s inflammatory processes. Recent studies have shown that PR3 impairs the resolution of inflammation and deregulates the immune system, contributing to the chronic nature of GPA. This deregulation is characterized by the activation of plasmacytoid dendritic cells and polarization of specific T-helper cells, which perpetuate the inflammatory cycle and lead to the characteristic granulomatous lesions seen in GPA.^[11]^

The latest criteria for the diagnosis of GPA are based mainly on the 2022 American College of Rheumatology/European Alliance of Associations for Rheumatology classification criteria for microscopic polyangiitis.^[12]^ According to the clinical criteria, patients with PR3-ANCA (or C-ANCA) positivity had the highest score of 5, and nasal involvement was followed by 3. The clinical criterion was assigned a score of 2, which included pulmonary manifestations, biopsies (granuloma, granulomatous inflammation, or giant cells), or cartilaginous involvement.

The key to obtaining the correct diagnosis of this case is recognizing that the patient has typical triad lesions of the upper respiratory tract and lung and kidney lesions. The patient had hematuria, for which tuberculosis, bacteria, or viruses could be ruled out. Therefore, the tuberculin skin test and metagenomic next-generation sequencing of skin herpes exudates would not be helpful. She had abdominal skin herpes and developed fever, which ruled out IgG4-related pulmonary disease and Behcet disease with pulmonary lesions.^[13,14]^ Therefore, the serum IgG4 concentration test, intradermal pathergy test or femoral vein thickness measurement would not be helpful. Her eye and nose lesions ruled out IgA nephropathy and anaphylactoid purpura.^[15,16]^ Therefore, qualitative urinary protein analysis and testing of inclusion and exclusion of the diagnosis of anaphylactoid purpura would not be helpful. Furthermore, previous literature indicates that the role of imaging diagnosis in this kind of pulmonary shadow is also limited.^[17,18]^

The emphasis in this disease is on the treating by anti-neutrophil cytoplasmic antibodies. The combination of glucocorticoids and cyclophosphamide is the preferred standard therapy for GPA.^[19]^ For young patients, cyclophosphamide should be replaced with azathioprine in maintenance therapy.^[19]^ Molecular targeted drugs (rituximab, especially) and plasmapheresis are also effective in the treatment of this disease.^[20]^

In general, GPA can be considered either a limited or a severe disease depending on its clinical presentation. It is generally not considered life-threatening for individuals with a limited GPA to suffer from symptoms that are focused primarily on the upper and lower respiratory tracts. Multisystem manifestations are common in people with severe diseases. When the kidneys are involved, the survival rate is 40%, and without kidney involvement, the survival rate is 60% to 70%.^[19]^ The diagnosis and treatment of GPA require the ability of a multidisciplinary physician to identify the disease. Otolaryngologists play a decisive role in recognizing the early onset of the disease and initiating appropriate treatment.^[19]^

## 4. Limitations

This case report analyses the skin herpes and rapidly developing lesions in multiple organs details of GPA and does not imply that all patients with GPA have these clinical signs and symptoms. Furthermore, the relevant pathophysiology mechanisms that cause these clinical symptoms have not been clarified.

## 5. Conclusion

In conclusion, this is the classic granulomatosis with polyangitis discovered from skin herpes and characterized by rapid lesions in multiple organs including eyes, nose, respiratory tract, lungs, and kidney. This case provides a vision of clinical diagnosis and treatment for chest diseases with non-chest symptoms.

## Acknowledgments

The authors wish to thank Yu Zhang, Peng Liu, and Biao Wang for their contributions during the discussion the diagnosis of the disease, data collection, and participation in the writing of the manuscript.

## Author contributions

**Conceptualization:** Naishi Wu, Wen-Juan Liao.

**Data curation:** Shu-Qing Ma.

**Formal analysis:** Jiantao Wu, Qu-Cheng Huang, Wen-Juan Liao.

**Funding acquisition:** Jiantao Wu, Wen-Juan Liao.

**Investigation:** Naishi Wu, Wen-Juan Liao.

**Methodology:** Shu-Qing Ma, Jiantao Wu, Naishi Wu, Wen-Juan Liao.

**Project administration:** Wen-Juan Liao.

**Resources:** Naishi Wu, Qu-Cheng Huang, Wen-Juan Liao.

**Software:** Hanlong Guo, Wen-Juan Liao.

**Validation:** Wen-Juan Liao.

**Visualization:** Hanlong Guo, Wen-Juan Liao.

**Writing – original draft:** Shu-Qing Ma.

**Writing – review & editing:** Jiantao Wu, Naishi Wu, Qu-Cheng Huang, Wen-Juan Liao.
